# Supplementary material for: Differential Responses of Transplanted Stem Cells to Diseased Environment Unveiled by a Molecular NIR-II Cell Tracker
Source: Research (Wash D C). 2021 Jun 19;2021:9798580. doi: 10.34133/2021/9798580 (PMC8237598; doi:10.34133/2021/9798580)
Supplement: Supplementary 1 — Figure S1: MALDI-TOF-MS spectrum of HAS. Figure S2: MALDI-TOF-MS spectrum of HSA-Tat. Figure S3: MALDI-TOF-MS spectrum of HSA-Tat/4T complex. Figure S4: (A) CelTrac1000 labeling concentration vs. iPSC-EC/MSC relative NIR-II fluorescent intensity. (B) CelTrac1000 iPSC-EC/MSC cytotoxicity analysis. Figure S5: comparison of gene expression between CelTrac1000-labeled iPSC-ECs (A) or MSC (B) and unlabeled control group. N = 3 per group. Figure S6: plot of CelTrac1000 fluorescent changes in PBS buffer at 37°C for 2 months. Figure S7: plot of integral fluorescent intensities at spot 5 (A, 15,625 iPSC-ECs) and spot 9 (B, 976 iPSC-ECs) postinjection. Figure S8: plot of the integral fluorescent intensities at spots 1-9 after 30 days postinjection. [file 9798580.f1.pdf]

Electronic Supporting Information

Differential Responses of Transplanted Stem Cells to Diseased Environment Unveiled by A Molecular NIR-II Cell Tracker

Hao Chen,[1,4]† Huaxiao Yang,[2]† Chen Zhang,[3] Si Chen,[4,5] Xin Zhao,[6] Mark Zhu,[4] Zhiming Wang,[1] Yuebing Wang,[4] Hung-Ta Wo,[6,7] Kai Li,[3]* and Zhen Cheng, [1,4]*

[1]Center for Molecular Imaging Research, Shanghai Institute of Materia Medica, Chinese Academy of Sciences, Shanghai, 201203, China

[2]University of North Texas, Biomedical Engineering, Denton, TX 76207

[3]Department of Biomedical Engineering, Southern University of Science and Technology, Shenzhen, Guangdong 518055, China

[4]Molecular Imaging Program at Stanford (MIPS), Bio-X Program, and Department of Radiology, Canary Center at Stanford for Cancer Early Detection, Stanford University, Stanford, California 94305-5344, United States[5]Department of Neurology, Xiangya Hospital, Central South University, Xiangya Road 88, Changsha, Hunan, 410008, China

[6]Stanford Cardiovascular Institute, Stanford, CA 94305, United States

[7]Division of Cardiology, Department of Internal Medicine, Chang Gung Memorial Hospital, Linkou, 33305, Taiwan

†These authors contributed equally

*Correspondence should be addressed to Kai Li; lik@sustech.edu.cn and Zhen Cheng; zcheng@stanford.edu

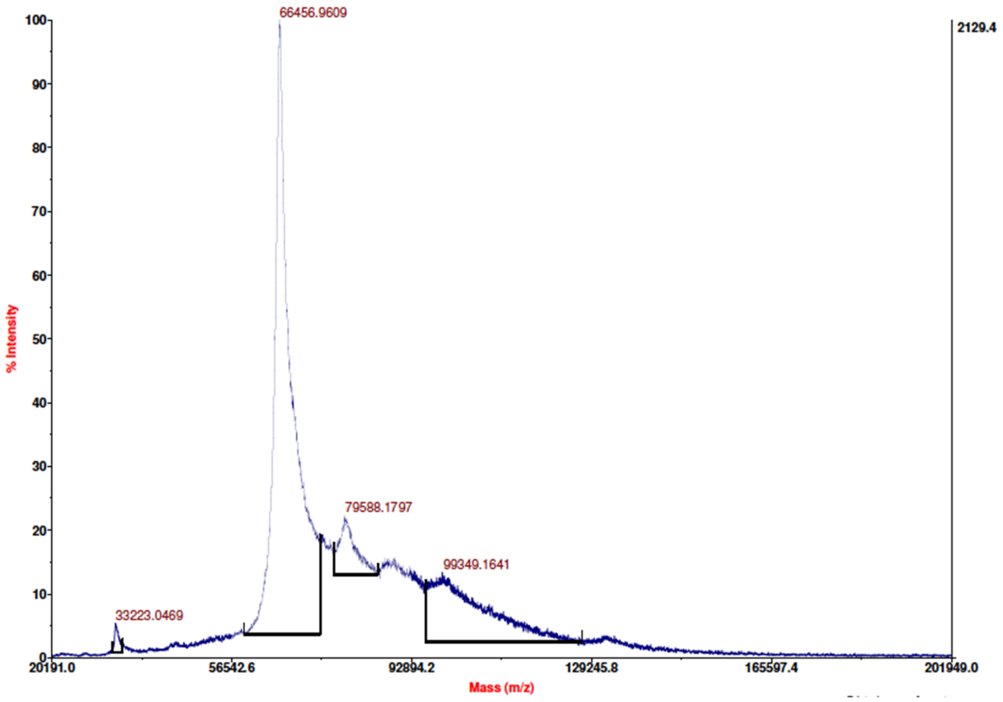

Figure S1. MALDI-TOF-MS spectrum of HSA.

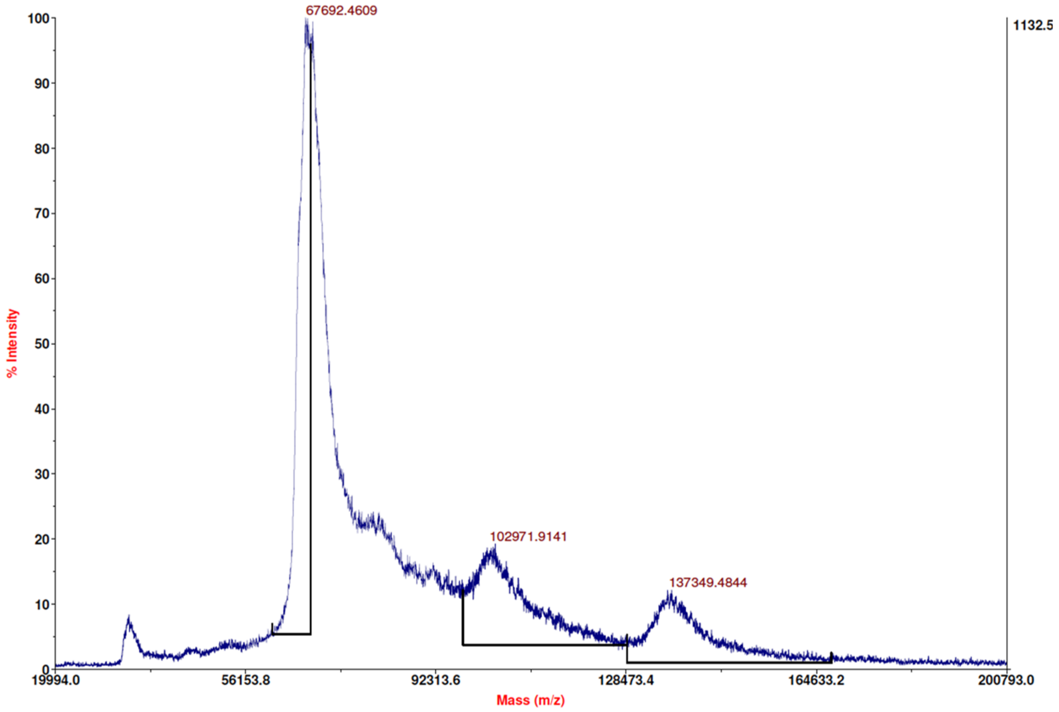

Figure S2. MALDI-TOF-MS spectrum of HSA-Tat.

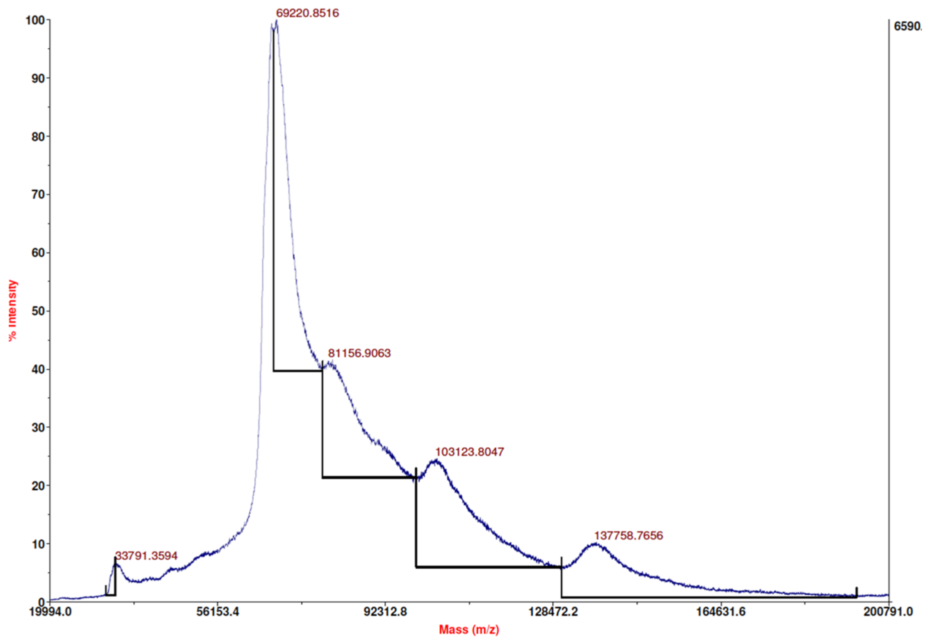

Figure S3. MALDI-TOF-MS spectrum of HSA-Tat/4T Complex.

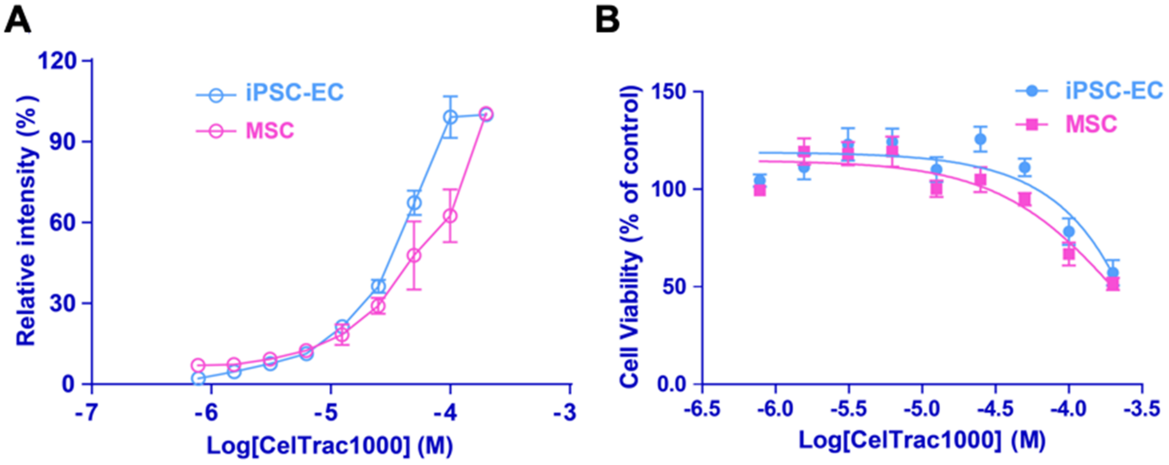

Figure S4. A) CelTrac1000 labeling concentration vs iPSC-ECs/MSCs relative NIR-II fluorescent intensity. B) CelTrac1000 iPSC-ECs/MSCs cytotoxicity analysis.

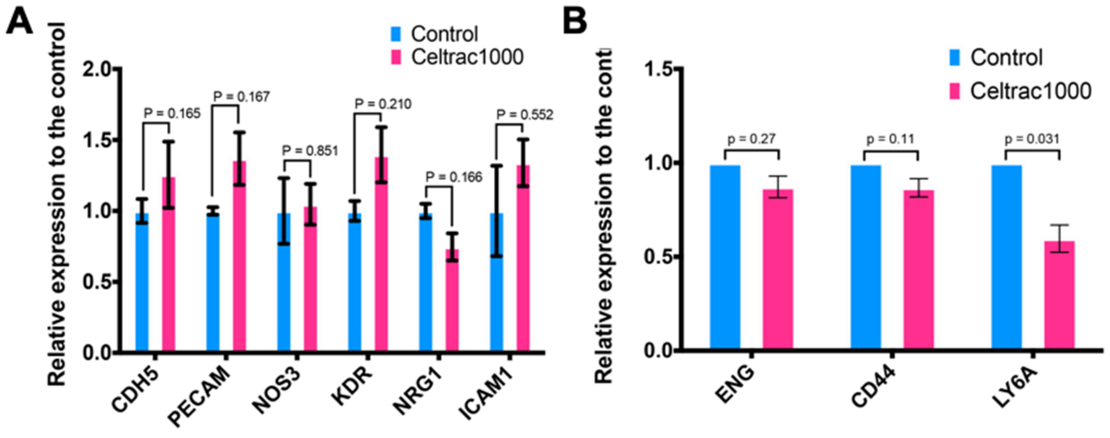

Figure S5. Comparison of gene expression between CelTrac1000 labeled iPSC-ECs (A) or MSC (B) and unlabeled control group. N = 3 per group.

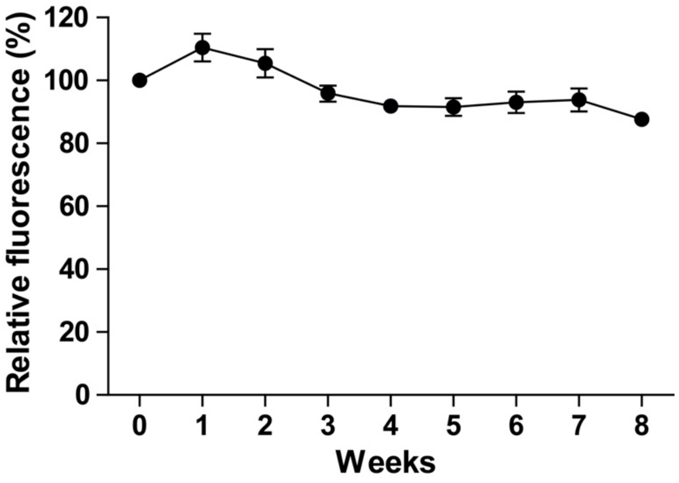

Figure S6. Plot of CelTrac1000 fluorescent changes in PBS buffer at 37 °C for 2 months.

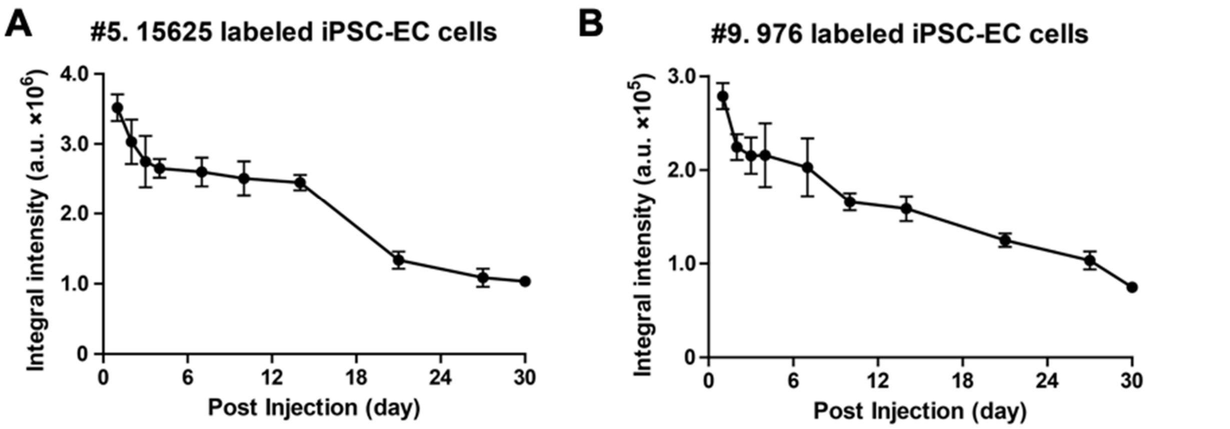

Figure S7. Plot of integral fluorescent intensities at spot 5 (A, 15,625 iPSC-ECs) and spot 9 (B, 976 iPSC-ECs) post injection.

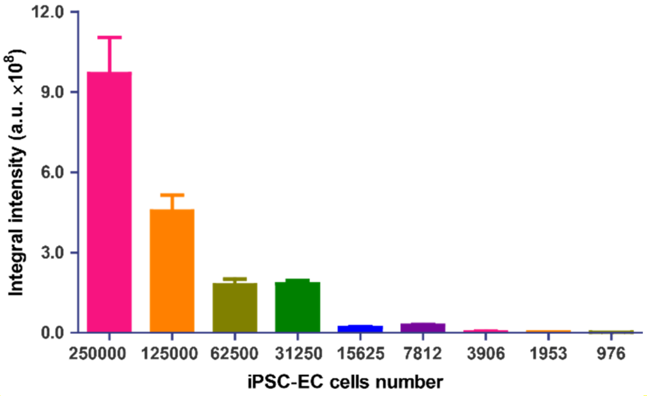

Figure S8. Plot of the integral fluorescent intensities at spot 1-9 after 30 days post injection.

Table S1. Advantages of CelTrac1000 in comparison to the current existing imaging techniques

**Techniques**	**Trackers**	**Pro & Con**
MRI, MPI Trackers	Gd^{3+}-, Mn^{2+}-, ^{19}F-based probes, iron oxide	Pros: Unlimited tissue penetration and good spatial resolution; Arbitrary orientation tomography and non-osseous artifacts; Long tracking period; Non-ionizing and low frequency magnetic fields
		Cons: Long scanning time and slow imaging speed
PET/SPECT Trackers	Radioisotopes-based materials: ^{18}F, ^{64}Cu, ^{89}Zr, ^{111}In, $^{86}Y/^{177}Lu$-based reagents	Pros: Good spatial resolution (1~2 mm); Variable tracking periods; Whole-body imaging with high sensitivity
		Cons: High cost; Ionizing radiation; Low temporal resolution
CT Trackers	Gold nanoparticles	Pros: Unlimited detection depth and good spatial resolution; Whole-body imaging with good sensitivity
		Cons: Ionizing radiation; *In vivo* excretion concern
NIR-I Fluorescent Trackers	Inorganic QDs, fluorescence nanodiamond, organic nanoparticles	Pros: Short acquisition time; Easy Operation; Low cost imaging system; High sensitivity without ionizing irradiation
		Cons: Limited imaging resolution and penetration depth (< 1 mm); Interference from biological auto-fluorescence
Previously reported NIR-II Fluorescent Trackers	Inorganic QDs	Pros: Short acquisition time; Deeper tissue penetration depth than NIR-I; Ultra low interference from biological auto-fluorescence
		Cons: Uncertain excretion of heavy metal; Only for animal studies
CelTrac1000	HSA-based molecular tracker	Pros: Share similar advantages with the NIR-II QDs; Excellent biocompatibility; Dynamic tracking of the migrating trajectory of administered cells in the mouse circulation system with a single cell cluster resolution
		Cons: Good performance in animal studies and preclinical applications, but only applicable in some scenarios for large animal and human

Video S1 (separate file). MSC clusters tracking video. 1100LP filer, 100ms exposure time, 5 mins post intravenous injection of CelTrac1000-labeled MSCs. Scale bar: 5 mm

Electronic Supporting Information

Video S2 (separate file). MSC clusters migration trajectory video. 1100LP filer, 100ms exposure time, 5 mins post intravenous injection of CelTrac1000-labeled MSCs. Scale bar: 5 mm

Video S3 (separate file). Mouse lung lobe movement video. 1100LP filer, 200ms exposure time, 1.5h post intravenous injection of CelTrac1000-labeled MSCs. Scale bar: 5 mm

Video S4 (separate file). Mouse heart beating video. 1000LP filer, 50ms exposure time, 0.5h post intravenous injection of CelTrac1000-labeled MSCs, heart in diastole and systole phases. Scale bar: 5 mm

Appendix:

1. The complete images of Fig. 4a

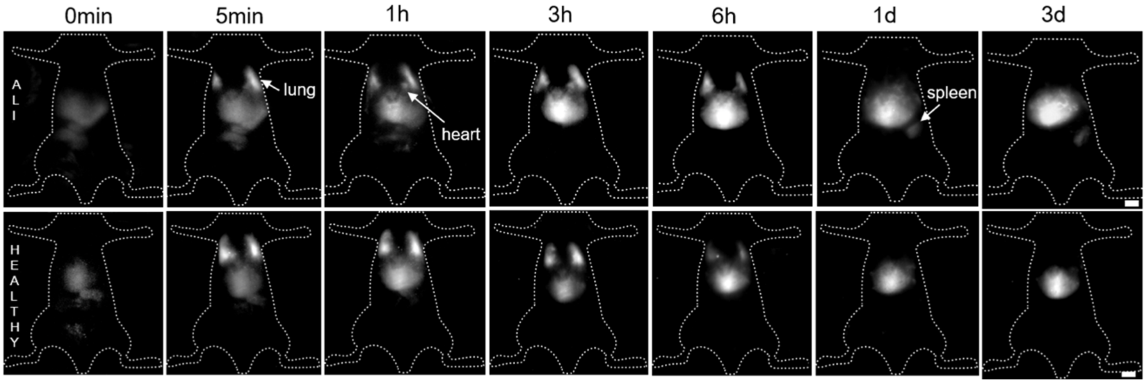

2. The complete images of Fig. 5a

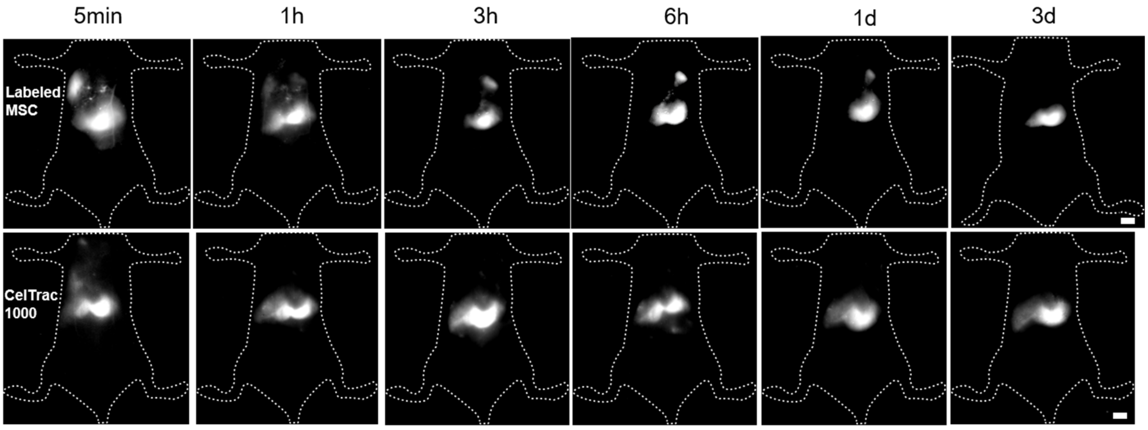

3. The calculation formulas of cellular uptake and leakage of CelTrac1000:
The cumulative amount of cellular uptake and leakage of CelTrac1000 MSCs and iPSC-ECs were calculated as follows:

Uptake:
first 24h

$$P_{n\,(uptake\ in\ cell)}\% = \frac{Mol_{n\,(in\ cell)}}{Mol_{(original)}} \times 100$$

$$= \frac{Mol_{n\,(in\ cell)} - [Mol_{n(in\ present\ medium)} + \sum_{i=1}^{n-1} Mol_{n-1(in\ taken\ out\ medium)}]}{Mol_{(original)}} \times 100$$

$$= 100 - \frac{[Mol_{n(in\,present\,medium)} + \sum_{i=1}^{n-1} Mol_{n-1(in\,taken\,out\,medium)}]}{Mol_{(original)}} \times 100$$

$$= 100 - \frac{\{[2mL - 0.1mL(n-1)]C_{n(in\,present\,medium)} + \sum_{i=1}^{n-1} Mol_{n-1(in\,taken\,out\,medium)}]}{Mol_{(original)}} \times 100$$

48h

$$P_{10\,(48h\,uptake\,in\,cell)}\% = 100 - \frac{\{2mL \times C_{n(in\,present\,medium)} + \sum_{i=1}^{n-1} Mol_{n-1(in\,taken\,out\,medium)}]}{Mol_{(original)}} \times 100$$

Where:

$$\sum_{i=1}^{n-1} Mol_{n-1(in\,taken\,out\,medium)}] = 0.1mL \times C_1 + 0.1mL \times C_2 + \cdots 0.1mL \times C_{(n-1)}$$

where P is the cumulative percentage of 4T/HSA-Tat in EC or MSC compare with the original, $Mol_{n\,(in\,cell)}$ is the nth mole amount of probe in cells, $Mol_{(original)}$ is the original mole amount of probe which was put into mediums, $Mol_{n(in\,present\,medium)}$ is the nth probe (mole amount) in present medium, $Mol_{n-1(in\,taken\,out\,medium)}$is the $(n-1)^{th}$ mole amount of the probe in taken out medium. C_n is the probe concentration of the nth taken out mediums (μmol/L).

Release:

$$P_{m\,(in\,cell\,after\,release)}\% = \frac{Mol_{(uptake\,in\,cell\,after\,48h)} - Mol_{(total\,release\,in\,medium)}}{Mol_{(original)}} \times 100$$

$$= \frac{Mol_{\,(uptake\,in\,cell\,after\,48h)} - [Mol_{m(in\,present\,medium)} + \sum_{i=1}^{m-1} Mol_{m-1(in\,taken\,out\,medium)}]}{Mol_{(original)}} \times 100$$

$$= P_{\,(uptake\,in\,cell\,after\,48h)} - \frac{[Mol_{m(in\,present\,medium)} + \sum_{i=1}^{m-1} Mol_{m-1(in\,taken\,out\,medium)}]}{Mol_{(original)}} \times 100$$

$$= P_{\,(uptake\,in\,cell\,after\,48h)} - \frac{\{[3mL - 0.1mL(m-1)]C_{m(in\,present\,medium)} + \sum_{i=1}^{m-1} Mol_{m-1(in\,taken\,out\,medium)}]}{Mol_{(original)}} \times 100$$

Where:

$$\sum_{i=1}^{m-1} Mol_{m-1(in\,taken\,out\,medium)}] = 0.1mL \times C_1 + 0.1mL \times C_2 + \cdots 0.1mL \times C_{(m-1)}$$

where P is the cumulative percentage of 4T/HSA-Tat in EC or MSC compare with the original, $Mol_{m(uptake\,in\,cell\,after\,48h)}$ is the mth mole amount of probe in cells, $Mol_{(original)}$ is the original mole amount of probe which was put into mediums, $Mol_{m(in\,present\,medium)}$ is the mth probe (mole amount) in present medium, $Mol_{m-1(in\,taken\,out\,medium)}$is the $(m-1)^{th}$ mole amount of the probe in taken out medium. C_m is the probe concentration of the mth taken out mediums (μmol/L).

Release with IPS treated:
First 6h

$$P_{l\,(in\,cell\,with\,IPS)}\% = \frac{Mol_{(in\,cell\,after\,48h\,release)} - Mol_{(total\,release\,in\,medium)}}{Mol_{(original)}} \times 100$$

$$= \frac{Mol_{\,(in\,cell\,after\,48h\,release)} - [Mol_{l(in\,present\,medium)} + \sum_{i=1}^{l-1} Mol_{l-1(in\,taken\,out\,medium)}]}{Mol_{(original)}} \times 100$$

$$= P_{\,(in\,cell\,after\,48h\,release)} - \frac{[Mol_{l(in\,present\,medium)} + \sum_{i=1}^{l-1} Mol_{l-1(in\,taken\,out\,medium)}]}{Mol_{(original)}} \times 100$$

$$= P_{\,(in\,cell\,after\,48h\,release)} - \frac{\{[3mL - 0.1mL(l-1)]C_{l(in\,present\,medium)} + \sum_{i=1}^{l-1} Mol_{l-1(in\,taken\,out\,medium)}]}{Mol_{(original)}} \times 100$$

Where:

$$\sum_{i=1}^{l-1} Mol_{l-1(in\,taken\,out\,medium)}] = 0.1mL \times C_1 + 0.1mL \times C_2 + \cdots 0.1mL \times C_{(l-1)}$$

where P is the cumulative percentage of 4T/HSA-Tat in EC or MSC compare with the original, $Mol_{l(in\,cell\,after\,48h\,release)}$ is the lth mole amount of probe in cells, $Mol_{(original)}$ is the original mole amount of probe which was put into mediums, $Mol_{l(in\,present\,medium)}$ is the lth probe (mole amount) in present medium, $Mol_{l-1(in\,taken\,out\,medium)}$is the (l-1)th mole amount of the probe in taken out medium. C_l is the probe concentration of the lth taken out mediums (μmol/L).

6h to 48h

$$P_{r\,(in\,cell\,after\,6h\,IPS)}\% = \frac{Mol_{(in\,cell\,after\,6h\,IPS)} - Mol_{(total\,release\,in\,medium)}}{Mol_{(original)}} \times 100$$

$$= \frac{Mol_{\,(in\,cell\,after\,6h\,IPS)} - [Mol_{r(in\,present\,medium)} + \sum_{i=1}^{r-1} Mol_{r-1(in\,taken\,out\,medium)}]}{Mol_{(original)}} \times 100$$

$$= P_{\,(in\,cell\,after\,6h\,IPS)} - \frac{[Mol_{r(in\,present\,medium)} + \sum_{i=1}^{r-1} Mol_{r-1(in\,taken\,out\,medium)}]}{Mol_{(original)}} \times 100$$

$$= P_{\,(in\,cell\,after\,6h\,IPS)} - \frac{\{[3mL - 0.1mL(r-1)]C_{r(in\,present\,medium)} + \sum_{i=1}^{r-1} Mol_{r-1(in\,taken\,out\,medium)}]}{Mol_{(original)}} \times 100$$

Where:

$$\sum_{i=1}^{r-1} Mol_{r-1(in\,taken\,out\,medium)}] = 0.1mL \times C_1 + 0.1mL \times C_2 + \cdots 0.1mL \times C_{(r-1)}$$

where P is the cumulative percentage of 4T/HSA-Tat in EC or MSC compare with the original, $Mol_{r\,(in\,cell\,after\,6h\,IPS)}$ is the rth mole amount of probe in cells, $Mol_{(original)}$ is the original mole amount of probe which was put into mediums, $Mol_{r(in\,present\,medium)}$ is the rth probe (mole amount) in present medium, $Mol_{r-1(in\,taken\,out\,medium)}$is the (r-1)th mole amount of the probe in taken out medium. C_r is the probe concentration of the rth taken out mediums (μmol/L).
